# Obesity and Dental Caries: A State-of-the-Art Review of Shared Risk Factors, Biological Mechanisms and Current Evidence

**DOI:** 10.3390/medsci14030336

**Published:** 2026-06-23

**Authors:** Inês Amaro, Anabela Paula, Ana Coelho, Carlos Miguel Marto, Mafalda Laranjo, Susana Alarico, Dírcea Rodrigues, Bárbara Oliveiros, Eunice Carrilho

**Affiliations:** 1 Institute of Integrated Clinical Practice, Faculty of Medicine, University of Coimbra, 3000-075 Coimbra, Portugal; appaula@fmed.uc.pt (A.P.); ana.coelho@uc.pt (A.C.); cmiguel.marto@uc.pt (C.M.M.); ecarrilho@fmed.uc.pt (E.C.); 2 Laboratory for Evidence-Based Sciences and Precision Dentistry, University of Coimbra, 3000-075 Coimbra, Portugal; 3 Coimbra Institute for Clinical and Biomedical Research (iCBR), Area of Environment Genetics and Oncobiology (CIMAGO), Faculty of Medicine, University of Coimbra, 3000-548 Coimbra, Portugal; uc42082@uc.pt (M.L.); boliveiros@fmed.uc.pt (B.O.); 4 Clinical Academic Center of Coimbra (CACC), 3004-561 Coimbra, Portugal; 5 Centre for Innovative Biomedicine and Biotechnology (CIBB), University of Coimbra, 3004-504 Coimbra, Portugal; salarico@cnc.uc.pt; 6 Centre for Mechanical Engineering, Materials and Processes (CEMMPRE), Advanced Production and Intelligent Systems (ARISE), Department of Mechanical Engineering, University of Coimbra, 3030-788 Coimbra, Portugal; 7 Institute of Experimental Pathology, Faculty of Medicine, University of Coimbra, 3000-548 Coimbra, Portugal; 8 Center for Neuroscience and Cell Biology (CNC), University of Coimbra, 3004-504 Coimbra, Portugal; 9 Department of Endocrinology, Diabetes and Metabolism, Unidade Local de Saúde de Coimbra (ULS Coimbra), 3004-561 Coimbra, Portugal; dircearodrigues@chuc.min-saude.pt; 10 Laboratory of Biostatistics and Medical Informatics (LBIM), Faculty of Medicine, University of Coimbra, 3000-548 Coimbra, Portugal

**Keywords:** obesity, dental caries, inflammation, risk factors, diet, oral hygiene, socioeconomic status, biomarkers

## Abstract

Obesity and dental caries are highly prevalent chronic conditions with significant global health impact. Although an association between these diseases has been suggested, the nature of this relationship remains unclear. This state-of-the-art review aims to synthesize current evidence on the interplay between obesity and dental caries, focusing on shared risk factors, salivary alterations and underlying biological mechanisms. Evidence indicates that obesity and dental caries share common behavioral and socioeconomic determinants, namely unhealthy dietary patterns with high intake of free sugars, poor oral hygiene habits and social disadvantage. Salivary alterations observed in obesity may also create a more cariogenic oral environment. Additionally, inflammatory mediators, oxidative stress markers and changes in the oral microbiome suggest biologically plausible links between both conditions. However, current data does not support a direct causal relationship, but rather a complex multifactorial interaction between obesity and dental caries driven by shared risk factors and modifiable behaviors. Preventive strategies should adopt an integrated approach targeting shared determinants, particularly diet, oral hygiene habits and socioeconomic status. Nevertheless, the predominance of cross-sectional evidence limits causal inference, highlighting the need for longitudinal studies that simultaneously assess obesity and dental caries, and that address salivary biomarkers using standardized methodologies across different age groups to clarify underlying mechanisms and assess their clinical relevance.

## 1. Introduction

Obesity is a chronic medical condition whose prevalence has increased dramatically over recent decades. It now stands as one of the most widespread non-communicable diseases globally and represents a major public health challenge [1].

According to the World Health Organization (WHO) [1], approximately 2.5 billion adults were overweight in 2022, including 890 million living with obesity. The pediatric population is equally affected; in 2024, 35 million children under the age of 5 were overweight, while 2022 data showed over 390 million individuals aged 5–19 were overweight, with 160 million of them being classified as obese. Projections of the World Obesity Federation [1] suggest that by 2035, the number of adults with a high Body Mass Index (BMI) will reach nearly 3.3 billion, with the figure of children and adolescents expected to exceed 770 million.

The WHO [2] defines obesity as a chronic disease characterized by abnormal or excessive fat accumulation resulting from an energy imbalance between calorie intake and expenditure. Diagnosis is typically based on the BMI, where individuals are categorized as overweight (BMI ≥ 25 kg/m^2^), obese (BMI ≥ 30 kg/m^2^) or morbidly obese (BMI ≥ 40 kg/m^2^) [3]. Obesity can be further categorized into three classes based on its severity, namely Class I (BMI 30.0–34.9 kg/m^2^), Class II (BMI 35.0–39.9 kg/m^2^) and Class III (BMI ≥ 40.0 kg/m^2^) [3,4].

Beyond its direct health impact, obesity is a complex, multifactorial condition influenced by genetic, behavioral, social and metabolic factors [5,6]. It is also a significant risk factor for numerous comorbidities, including cardiovascular disease, obstructive sleep apnea, type 2 diabetes *mellitus*, dyslipidemia, hypertension and various types of cancer [5,7].

Treatment of obesity requires a multidimensional approach combining lifestyle modification, behavioral interventions, pharmacotherapy and, in severe cases, bariatric surgery [8,9]. In pediatric populations, treatment options must consider growth, family context, and psychosocial factors, with multidisciplinary and family-based interventions proving most effective, alongside broader public health strategies [10,11].

While obesity is primarily managed as a systemic condition, its metabolic and behavioral consequences often extend to oral health, requiring an investigation into its potential relationship with dental caries [12].

Dental caries remains one of the most prevalent oral diseases worldwide, affecting all age groups and serving as the primary cause of toothache and tooth loss [13,14]. Data from the WHO indicates that dental caries affects an estimated 2 billion individuals with permanent dentition and over 510 million children with primary dentition [15].

It is a dynamic, initially reversible process driven by the interaction between the oral microbiome, dietary sugars and host-related factors such as salivary composition and flow rate [16,17]. When this balance is disrupted, the continuous loss of minerals from the tooth structure leads to cavity formation. Dental caries is, therefore, considered a biosocial disease, as its development is intrinsically linked to individual behaviors, socioeconomic context and lifestyle-related factors [13,14,16].

In recent years, a growing body of evidence has suggested a significant association between obesity and dental caries. Obesity-related metabolic disturbances can influence salivary characteristics and composition, favoring the development of cariogenic species [18]. Moreover, both conditions share several common risk factors, including high consumption of free sugars [19].

Furthermore, age significantly influences the prevalence and severity of both obesity and dental caries. Evidence suggests that the association between BMI and caries is more pronounced in the permanent dentition [20,21,22], likely because both conditions are chronic and cumulative, allowing their interaction to become more detectable as they progress throughout the life course [21,22,23].

Although pediatric populations remain the main focus of current research [12,24,25,26,27,28], extending this analysis to adults is equally important. Cumulative exposure to shared risk factors and obesity-related comorbidities may further compromise oral health. From a life-course perspective, both conditions are shaped by early-life exposures, behavioral patterns and long-term social determinants. However, current evidence in adults remains inconsistent, with contradictory findings [29,30,31,32,33].

Within the adult population, individuals undergoing bariatric surgery warrant particular attention. These individuals often face an exacerbated risk of dental caries due to increased meal frequency, prolonged exposure to fermentable carbohydrates [6,34] and reported increases in salivary *Streptococcus mutans* levels [35]. Postoperative complications, such as gastroesophageal reflux induced by rapid ingestion, inadequate mastication or recurrent vomiting, create an acidic oral environment that impairs salivary buffering capacity [6,36,37]. Additionally, a preference for soft, adhesive foods and a reduced intake of anticariogenic dairy products further heighten caries risk [6]. Nevertheless, integrated monitoring can effectively mitigate these oral complications [34].

In summary, the association between obesity and dental caries is not direct or uniform, but is instead mediated by complex interactions between dietary, behavioral, socioeconomic and biological determinants. Consequently, BMI should not be used as a standalone predictor of caries risk.

Although previous systematic reviews and meta-analyses have examined the association between obesity and dental caries, these studies have primarily focused on quantifying the epidemiological relationship through the association of BMI and caries index data, often within specific age groups. Recent reviews have also explored obesity or caries-related salivary biomarkers and inflammatory pathways, providing valuable insights into potential biological mechanisms. However, these studies have generally addressed obesity or oral health in isolation and have not comprehensively integrated the behavioral, socioeconomic, physicochemical, molecular, and microbial factors that may jointly contribute to the obesity-dental caries association. The present state-of-the-art review therefore aims to fill this gap by providing a broader and more integrative synthesis of the current evidence, with particular emphasis on the multifactorial interactions that may connect these conditions, providing a comprehensive, mechanistically oriented overview of the obesity-dental caries relationship. Ultimately, it seeks to highlight the importance of integrated health strategies for the prevention and management of these two global health challenges while identifying knowledge gaps that warrant further investigation.

## 2. Materials and Methods

A literature search was conducted in the electronic databases PubMed, Cochrane Library, Web of Science and Scopus. No language or publication date restrictions were applied, and the search was updated through April 2026.

The search strategy was developed using a combination of Medical Subject Headings (MeSH) terms and relevant keywords, including: “dental caries”, “obesity”, “overweight”, “body mass index”, “risk factors”, “diet”, “sugar”, “oral hygiene”, “toothbrushing”, “socioeconomic status”, “income”, “education”, “occupation”, “saliva”, “biomarker” and “microbiome”. The primary search strategy used was: (“dental caries” AND (obesity OR overweight OR “body mass index”)). Additional search combinations were developed according to the specific domains explored in this review, including lifestyle and dietary habits, oral hygiene habits, socioeconomic status, and salivary alterations. Given the narrative state-of-the-art nature of this review, search terms and combinations were applied iteratively and adapted according to the specific thematic domains explored, rather than following a predefined systematic search protocol.

To maximize retrieval of relevant literature, the reference lists of all eligible articles were manually screened for additional studies not identified through the database search. The study selection process was independently performed by two reviewers and any discrepancies were resolved through discussion and consensus. The included studies were subsequently reviewed in full, categorized according to the main topics addressed and critically appraised. Evidence was synthesized narratively to provide an integrated overview of the current knowledge regarding the relationship between obesity and dental caries.

Clinical, epidemiological and experimental studies investigating dental caries and/or obesity, as well as their potential association with behavioral, socioeconomic, salivary, inflammatory and microbiological determinants were considered for inclusion. Studies which failed to report objective measures of obesity (e.g., BMI, BMI z-score, waist circumference, or obesity classification), did not assess dental caries using recognized clinical or epidemiological measures (e.g., DMFT/dmft, ICDAS, caries prevalence), or did not provide sufficient quantitative or qualitative relevant data regarding obesity, dental caries or their potential association, shared risk factors, salivary characteristics, or biological mechanisms were excluded.

As this work was designed as a state-of-the-art narrative review aimed at providing a broad and integrative synthesis of the available evidence, no formal quality assessment tool was applied. Nevertheless, the methodological strengths and limitations of the included studies were critically discussed throughout the manuscript.

## 3. Multifactorial Etiology—Risk Factors

Both obesity and dental caries arise from a complex, multifactorial etiology and share several common risk factors. However, their relationship remains controversial, as neither condition is a definitive predictor of the other. To understand the conflicting findings in current literature, these conditions must be evaluated alongside contributing host-related determinants, including socioeconomic status (SES), education, lifestyle and oral hygiene (OH) habits [24]. Failure to account for these variables limits study comparability and inhibits the establishment of definitive causal pathways.

Consequently, a critical appraisal of shared behavioral, socioeconomic and biological factors is essential to address this heterogeneity, with dietary habits representing the most consistent and biologically plausible link.

### 3.1. Lifestyle and Dietary Habits

Modern lifestyle factors, such as increased screen time and the pervasive marketing of ultra-processed, sugar-rich foods via social media, promote sedentary behavior and frequent snacking [20,38,39,40,41]. These environmental influences play a contributory role in shaping high-risk dietary habits, although they likely reflect broader lifestyle patterns rather than a direct causal mechanism. Conversely, regular physical activity is often associated with healthier lifestyle behaviors, including improved nutritional choices and better oral hygiene, which collectively reduce caries prevalence [42,43].

The rising prevalence of both conditions is largely driven by unhealthy dietary patterns characterized by the high availability of energy-dense, processed foods [22,44,45,46]. Free sugars, particularly sucrose and glucose, are central to this association as they promote excess adiposity while simultaneously fostering an acidic oral environment that favors cariogenic bacteria [40]. Beyond simple sugars, fermentable carbohydrates such as starches also contribute to this risk [47]. When broken down in the oral cavity, starches lead to sugar accumulation on tooth surfaces, lowering oral pH and facilitating enamel demineralization [47].

Evidence consistently links higher BMI and Decayed, Missing and Filled Teeth (DMFT/dmft) index scores to the frequent consumption of sugar-sweetened beverages (SSB), fast foods and processed snacks, while negative associations are observed with the intake of fruits, vegetables and proteins [39,48]. While some traditional staples like milk may have protective properties, these benefits are often offset when beverages are sweetened with added sugars [49]. However, the strength of these associations may vary across studies, partly due to differences in dietary assessment methods, age groups—as most of these studies are conducted in children—and adjustment for socioeconomic and behavioral confounders. Moreover, dietary intake is frequently self-reported, increasing the risk of measurement and recall bias.

Furthermore, the frequency and timing of intake are critical. Snacking between meals and sugar consumption before bedtime are particularly hazardous as reduced salivary flow during sleep impairs sugar clearance and prolongs acidic conditions, exacerbating demineralization [33,39,47,50,51,52]. Also, beyond their direct effects on the oral environment, obesity-related salivary alterations may also influence masticatory function and oral health outcomes, with potential consequences for dietary behaviors.

Salivary and masticatory alterations may represent potential biological pathways linking obesity and dental caries. Obesity is associated with impaired salivary parameters, including reduced flow rate, pH and buffering capacity, all of which correlate with higher caries experience [53,54]. Besides dental caries, obese adult individuals might also present more missing teeth compared to normal-weight individuals [23,45,55]. Tooth loss has a significant negative impact on quality of life, leading to impaired chewing ability, inadequate dietary intake and functional limitations, which may further contribute to obesity by reducing masticatory function [56,57]. Conversely, obesity is also identified as a risk factor for tooth loss as it is associated with chronic low-grade systemic inflammation state, which may impair immune function and increase susceptibility to periodontal disease, a major cause of tooth loss [57].

Therefore, this relationship may be bidirectional: obesity-induced systemic inflammation and dietary habits can promote caries and tooth loss and, conversely, dental pain and reduced masticatory efficiency may lead individuals to prefer soft, energy-dense and easily ingested foods, further contributing to weight gain [23,57,58]. This cycle is often reinforced by reduced bite force [59] and impaired masticatory function, which can delay satiety and increase overall caloric intake [56]. However, it should be acknowledged that the evidence supporting this bidirectional model is derived predominantly from cross-sectional studies, limiting causal inference. Furthermore, the observed associations are likely influenced by multiple confounding factors, including age, socioeconomic status, smoking habits, chronic health conditions and access to dental care, which are not always controlled for simultaneously in the available literature. Although a bidirectional relationship has been hypothesized, longitudinal data examining whether masticatory impairment prospectively contributes to weight gain or whether obesity-related inflammation precedes tooth loss remain scarce. Therefore, the clinical significance and temporal direction of this association have yet to be established through adequately designed prospective cohort studies.

Dietary behaviors do not occur in isolation but are influenced by emotional regulation and taste perception [40]. Individuals experiencing stress or low mood—conditions more prevalent among obese populations [60]—often seek highly palatable, sugar-rich foods that activate reward pathways, a behavior that contributes to obesity while also increasing oral bacterial activity, lowering pH and promoting enamel demineralization [40,61]. Additionally, obesity-related physiological changes may alter taste perception [62], potentially driving the consumption of more energy-dense foods [63].

The domestic environment is also a primary determinant of health trajectories. Maternal factors, particularly BMI and educational level, strongly influence children’s eating habits and caries risk [64,65,66,67]. Higher maternal education typically correlates with better adherence to nutritional guidelines, whereas lower educational attainment is often a predictor of higher SSB intake and limited access to healthy food options [66,67,68,69]. Furthermore, as children gain autonomy, reduced parental supervision can lead to increased sugar intake. In fact, evidence from a recent systematic review [70] suggests that early-life dietary trajectories influence both dental caries and obesity, with high sugar intake increasing caries risk and poor dietary patterns contributing to greater adiposity later in life. These findings highlight the importance of establishing healthy dietary habits from infancy, as early nutritional exposures may have long-lasting effects on both oral and general health.

While the family remains foundational, the school environment also plays a vital role. School-based interventions and vending machine regulations have shown promise in reducing SSB consumption, especially when involving family participation [71,72].

Although diet appears to be a central shared determinant, its effect on caries risk is likely modified by protective behaviors, particularly OH practices.

### 3.2. Oral Hygiene Habits

Effective OH serves as a critical modifier in the relationship between diet, obesity and dental caries. While a cariogenic diet is a primary driver of decay, regular toothbrushing can partially mitigate these effects. In fact, evidence consistently demonstrates a robust inverse relationship between toothbrushing frequency and caries prevalence, with the effect being more pronounced in the deciduous than in the permanent dentition [73], whereas the combination of inadequate plaque control and high sugar intake synergistically exacerbates caries risk [74].

Adherence to oral health guidelines—specifically brushing at least twice daily with fluoride toothpaste—remains the gold standard for prevention [75]. Fluoride provides essential protection through topical mechanisms and its consistent use is significantly associated with lower DMFT values [17,76,77,78].

OH behaviors are often reflective of general health-related practices [79]. Beyond the direct mechanical removal of plaque, several biological pathways have been proposed to link OH and obesity. Poor OH triggers local inflammation, which can elevate systemic levels of C-reactive protein, a recognized marker of obesity [79,80]. Furthermore, subgingival plaque accumulation may contribute to systemic metabolic changes via endotoxemia. Lipopolysaccharide-producing bacteria can enter the bloodstream, potentially influencing adipose tissue accumulation [81]. Consequently, poor toothbrushing frequency has been associated with both higher BMI and increased caries prevalence, which may reflect a potential interplay between systemic inflammation and oral health behaviors, although the underlying mechanisms remain to be fully elucidated. Notably, some studies [20,23,53,79,81,82,83,84,85] suggest that oral hygiene practices may partially mediate the relationship between obesity-related salivary alterations and caries outcomes, as statistical adjustment for hygiene variables has been reported to attenuate this association. However, it should be noted that the available evidence is still largely derived from observational studies, and robust quantitative evidence addressing this association remains limited. Furthermore, considerable heterogeneity in the assessment of OH behaviors and caries outcomes limits direct comparisons across studies.

Despite the clear benefits of preventive practices, individuals with obesity are frequently reported to have irregular dental attendance [79]. This pattern may be partly attributed to weight-related stigma, which often leads to the avoidance of healthcare settings [86]. Moreover, socioeconomic barriers, including low income, lack of insurance, limited health literacy and transportation challenges, further restrict access to regular dental visits, exacerbating oral health inequalities [87]. Higher educational attainment and SES are consistently linked to superior OH practices and fewer decayed teeth [45]. Conversely, caries risk is highest when poor OH coexists with low SES, although proactive hygiene behaviors can partially offset these structural disadvantages [88].

Taken together, OH appears to be an important modifiable factor in the obesity-caries relationship. Nevertheless, the available evidence remains mainly observational, and the extent to which OH independently influences this association, rather than acting as a marker of broader behavioral and socioeconomic determinants, remains uncertain.

Ultimately, both dietary choices and OH behaviors are deeply embedded within a broader social and economic framework. These determinants not only shape individual habits but also explain the disproportionate distribution of obesity and dental caries across different population strata.

### 3.3. Socioeconomic Status

Socioeconomic factors significantly influence the prevalence of dental caries among obese populations, although reported findings remain inconsistent. Rather than a mere background characteristic, socioeconomic position acts as a determinant of exposure, susceptibility, and access to preventive care for both conditions.

SES is typically assessed through three distinct but interrelated dimensions: education, occupation, and income, each affecting health through different pathways [89]. While education dictates health literacy, occupation reflects social and environmental exposures, and income determines the material capacity to access health-related resources [89,90].

Historically, obesity was more prevalent among higher socioeconomic strata. However, this pattern has shifted in middle and high-income countries, where lower SES now bears a disproportionate burden of both obesity and dental caries [89,91].

Low income and food insecurity often lead to “nutritional poverty,” where nutrient-rich foods are replaced by more affordable, energy-dense alternatives high in fats and free sugars [26,89,92]. This is frequently exacerbated by environmental factors in disadvantaged neighborhoods, such as a high density of fast-food outlets and limited access to fresh produce [92,93,94]. In terms of oral health, food insecurity may lead to frequent and irregular consumption of low-quality snacks to extend food availability, thereby increasing the frequency of acid challenges to the enamel and elevating caries risk [26,93,94]. Nevertheless, the relationship between food insecurity and obesity is complex and may vary according to age, sex, cultural context, and national food environments, contributing to some of the inconsistencies reported in the literature.

Among the various dimensions of SES, educational attainment has been consistently identified as one of the strongest predictors of long-term health outcomes [95]. In pediatric populations, higher maternal educational levels are consistently associated with a lower risk of dental caries and obesity, reflecting superior health literacy and the adoption of protective behaviors, such as supervised toothbrushing and regulated dietary habits [17,69,96,97]. Conversely, lower educational attainment in adults is significantly linked to higher DMFT scores and missing teeth [45,98]. Interestingly, individuals with higher SES often present a higher number of filled teeth, reflecting a greater financial and cognitive capacity to seek and receive restorative dental care [39,45]. However, as previously stated, education is closely linked to other factors, such as health literacy, preventive healthcare use, and health-related behaviors, making it difficult to determine whether the observed associations are driven by education itself or by these related socioeconomic influences.

Occupation further reflects these inequalities, with less specialized roles often associated with higher stress, lower job control, and limited resources, factors linked to increased BMI [89,95]. These effects often manifest differently across sexes [95]. The association between lower SES and obesity appears to be stronger among women, reflecting the complex interaction between socioeconomic inequalities, limited career progression, and gender-related social roles, such as the cumulative burden of caregiving responsibilities [87,89,95]. Additionally, women may be more susceptible to sociocultural pressures and weight-related stigma, which further strengthens the link between social disadvantage and metabolic health [89,90,95].

While children in some industrialized settings may still show a positive correlation between household income and obesity due to sedentary lifestyles and easy access to sugary foods [24,25,56,88], the overarching global trend points toward a concentration of both conditions in socioeconomically vulnerable groups. These contrasting findings underscore the necessity of a comprehensive assessment of both country-level and individual socioeconomic factors since these discrepancies may reflect differences in economic development, cultural norms, dietary transitions, and healthcare systems, highlighting the context-dependent nature of the association between socioeconomic status, obesity, and dental caries.

Overall, socioeconomic disadvantage appears to be a consistent shared determinant of obesity and dental caries. However, the available evidence is predominantly observational, and the complex interplay between socioeconomic, behavioral, environmental, and biological factors limits the ability to establish direct causal pathways. Beyond these behavioral and social determinants, attention has increasingly turned to biological mechanisms that may help explain why some individuals with obesity are more susceptible to dental caries than others.

## 4. Salivary Alterations

The behavioral and socioeconomic determinants described do not act exclusively through direct exposure to cariogenic substrates as they also shape the biological environment of the oral cavity itself. Among these biological mediators is saliva, a complex biological fluid essential for maintaining oral homeostasis. As a clear, exocrine secretion with a slightly acidic pH (6.6–7.1), it comprises water (94–99%) and a diverse array of organic and inorganic components that facilitate digestion, lubrication, and the preservation of dental integrity [99,100,101]. As the primary defense system of the oral cavity, saliva protects tooth structures from demineralization and actively promotes remineralization [102,103].

Evaluating salivary parameters is crucial when investigating the obesity-caries relationship, as obesity-related metabolic shifts can impair salivary synthesis, composition and secretion [104,105]. Obesity is frequently associated with alterations in physicochemical properties, such as pH, buffering capacity, and flow rate, which may disrupt the oral microbiome and heighten caries risk [18,106]. These changes often reflect systemic disturbances, including insulin resistance and chronic inflammation, positioning saliva as a valuable non-invasive biomarker of systemic health [107]. This relationship can be examined through three lenses: physicochemical properties, molecular composition and microbial profiles.

### 4.1. Physicochemical Properties

Salivary flow rate is a critical determinant of oral protection. Saliva is classified as either unstimulated or stimulated, with both forms differing significantly in flow rate and biochemical composition [101,103].

Adequate flow facilitates the mechanical cleansing of the oral cavity and ensures the availability of bicarbonate ions for acid neutralization [108]. Conversely, hyposalivation impairs the clearance of fermentable carbohydrates and microorganisms, promoting biofilm accumulation [108,109].

Obesity is consistently associated with reduced salivary flow rates [12,18,106], both in children and adults, potentially due to proposed mechanisms including chronic inflammation, adipocyte infiltration of the salivary glands, and autonomic nervous system dysfunction, although direct evidence supporting these pathways remains limited. Furthermore, pharmacological management of obesity-related comorbidities such as hypertension, type 2 diabetes *mellitus* and mental health disorders, often induces xerostomia as a side effect [60,106,110]. Notably, emerging treatments like glucagon-like peptide-1 (GLP-1) receptor agonists (e.g., semaglutide) may further impair salivary function [111]. However, it should be taken into consideration that the widespread use of medications among obese individuals may act as an important confounding factor, making it difficult to distinguish obesity-related salivary alterations from treatment-related effects.

Under physiological conditions, salivary pH remains near neutral. When bacterial metabolism lowers plaque pH below the critical threshold (~5.5), demineralization occurs [16,108]. Obese individuals frequently exhibit lower unstimulated salivary pH, likely reflecting baseline oral conditions compromised by glandular hypofunction [16,18,40,106,108]. This reduction in pH, combined with the diminished buffering capacity, creates a synergistic environment that favors the proliferation of aciduric, cariogenic bacteria [18,54,108,109,112,113,114].

Although these physicochemical changes may create a more cariogenic oral environment, they do not fully explain the biological complexity of the association, which has prompted growing interest in salivary molecular markers.

### 4.2. Molecular Composition

Given that teeth are continuously exposed to saliva, its molecular constituents exert a significant influence on the initiation and progression of dental caries [115]. Current literature identifies several molecules, including tumor necrosis factor-alpha (TNF-α), alpha-amylase (α-amylase), total antioxidant capacity (TAC) and specific interleukins (IL), namely IL-6 and IL-8, as promising salivary biomarkers for the diagnosis and early detection of dental caries [18,100,102,115,116].

In the context of obesity, while biomarkers are traditionally measured in serum, many are detectable in saliva. This suggests that saliva may serve as a non-invasive, cost-effective alternative for diagnostic and monitoring purposes [117,118].

Among these constituents, inflammatory cytokines and adipokines have received particular attention, as they reflect both local oral inflammation activity and systemic metabolic imbalances.

Obesity is characterized by chronic low-grade inflammation, driven by inflammatory cell infiltration into adipose tissue and the increased production of cytokines and adipokines [107]. Similarly, dental caries involves bacterial-induced demineralization that triggers the release of inflammatory mediators, which regulate innate and adaptive immune responses [100].

As a key pro-inflammatory mediator secreted by adipocytes, TNF-α promotes inflammatory responses by generating reactive oxygen species and activating multiple signaling pathways [117]. Evidence indicates that individuals with obesity exhibit higher salivary TNF-α levels than those of normal weight [107,117], likely due to positive feedback mechanisms in adipose tissue [107]. Similarly, recent meta-analysis reported significantly higher TNF-α levels in individuals with active dental caries compared with caries-free controls [18,100]. Interestingly, although elevated TNF-α levels have been reported in both obesity and dental caries [119], the limited number of studies assessing these conditions simultaneously, together with predominantly cross-sectional designs, limits definitive conclusions regarding potential interactions between them.

IL-6 also plays a fundamental role in the pathophysiology of obesity, promoting macrophage infiltration into adipose tissue and contributing to a systemic pro-inflammatory state [117,118,120]. Even though there are recent reviews [117,118,120] linking elevated salivary IL-6 concentrations in obese individuals, most of the available studies on this matter remain of cross-sectional design and mainly focused on pediatric populations, with only a limited number of studies addressing adults. In the carious process, IL-6 is the most prevalent cytokine in the saliva of children and young adults with active disease, according to a recent quantitative synthesis [100]. Although IL-6 has been extensively investigated in relation to dental caries and there is moderate evidence regarding its association with obesity, evidence addressing both conditions simultaneously [82,121,122] remains scarce and methodologically diverse.

IL-8 is primarily produced by visceral adipocytes and regulates and amplifies inflammatory responses and immune cell recruitment [117,118]. While a tendency toward higher salivary IL-8 levels in obese individuals has been reported, results remain inconsistent [117]. Regarding dental caries, although meta-analysis showed higher IL-8 levels in children and young adults with caries lesions, these differences often lack statistical significance [100]. Evidence evaluating IL-8 in the context of both obesity and dental caries simultaneously is scarce and largely limited to the pediatric population, with some findings contrasting typical caries literature [121]. Although the authors raise the possibility that obesity and dental caries may influence salivary IL-8 levels through distinct or overlapping pathways, the available evidence remains limited and heterogeneous, preventing definitive conclusions regarding potential shared mechanisms or the clinical applicability of IL-8 as a biomarker.

Beyond inflammatory mediators, other host defense components, such as Immunoglobulin A (IgA), salivary α-amylase (sAA) and TAC, may influence caries susceptibility in obese individuals.

Salivary IgA is an anti-inflammatory immunoglobulin that plays a complex role in oral immunity. Despite its biological relevance, the current evidence supporting its role as a biomarker in the obesity-dental caries relationship remains heterogeneous and inconclusive. In the context of dental caries, some studies have reported reactive increases in IgA levels in response to bacterial challenges [123], whereas others have associated lower IgA concentrations with greater caries susceptibility [105]. This duality stems from IgA’s ability to both neutralize pathogens and potentially facilitate bacterial adhesion to tooth surfaces [100,105]. In obesity, IgA has been associated with chronic stress response activation [120], although the available evidence is still limited with studies in children and adolescents reporting contradictory findings [124,125,126]. Furthermore, studies evaluating IgA in the context of both obesity and dental caries simultaneously are scarce and predominantly pediatric-focused. Although one study [127] suggested a stronger association between IgA levels and dental caries than with obesity, the limited number of available evidence prevents definitive conclusions regarding the relative influence of either condition.

As the most prevalent salivary enzyme, sAA aids in carbohydrate digestion and modulates bacterial adhesion through its interaction with oral *Streptococci* [128]. While it increases substrate availability for cariogenic bacteria, it also binds to microorganisms to facilitate their clearance [128]. Consequently, both positive and inverse associations with dental caries have been reported in a recent meta-analysis, studying mostly children and young adults [129]. In obese populations, evidence is inconsistent among age groups [126,130,131,132,133], though some research indicates a positive correlation between sAA and DMFT in overweight adolescents, warranting further investigation [133].

TAC reflects the integrated action of enzymes and dietary antioxidants against oxidative damage [134]. However, paradoxically, a recent meta-analysis [18] found that unstimulated salivary TAC is elevated in both caries-active and overweight/obese individuals. This elevation may represent a compensatory antioxidant response to increased reactive oxygen species in the early stages of obesity rather than a reduction in defenses [134,135,136,137]. Similar to IgA and sAA, the current evidence supporting TAC as a clinically useful biomarker remains limited and inconclusive.

Taken together, TNF-α and IL-6 currently represent the most promising salivary biomarkers linking obesity and dental caries, although the evidence remains insufficient to establish their clinical applicability or to clarify their role in the interplay between both conditions. In fact, the overall evidence linking salivary biomarkers to the obesity-caries intersection remains limited and inconclusive. The dual and context-dependent roles of markers such as IgA and sAA, combined with the scarce and heterogeneous nature of available studies, namely differences in study design, sample sizes, participant characteristics, saliva collection and storage protocols and biomarker quantification methods, inhibit the establishment of causal relationships. Importantly, while moderate evidence supports the relevance of these markers when obesity or dental caries are examined in isolation, studies simultaneously evaluating both conditions and their potential synergistic effects on biomarker levels are scarce and predominantly cross-sectional, preventing any inference regarding the temporal sequence of biomarker changes relative to the onset or progression of either condition. A further limitation concerns the generalizability of current findings as a substantial proportion of the available evidence derives from pediatric populations, and extrapolation to adolescents and adults is not straightforward given the age-related differences in salivary composition, immune function and caries risk dynamics. Taken together, although several salivary biomarkers show investigational promise, their clinical applicability remains limited, their translational potential should be interpreted with caution and age-stratified prospective studies, designed to simultaneously assess both conditions across the life course and benchmarked against established systemic biomarkers, are needed before any biomarker-based conclusions can be broadly generalized. In addition, other biomarkers, including IL-15, IL-10 and IL-1β, have also been explored but remain poorly understood [118,120,121,138,139,140]. Taken together, these biochemical and immunological alterations may also influence the ecological balance of the oral cavity. Understanding how these biochemical shifts translate into changes in the oral microbiome is therefore a necessary step toward a complete mechanistic understanding of the obesity-caries relationship.

### 4.3. Microbial Profile

Saliva harbors a diverse microbial community that varies according to age, sex, and physiological status. Salivary parameters, particularly flow rate and pH, play a critical role in shaping the composition and metabolic activity of the oral microbiome [141], which can undergo significant modifications under pathological conditions such as obesity and dental caries. From this perspective, the oral microbiome may represent a potential mediator of the association between obesity-related metabolic shifts and the dysbiotic processes involved in dental caries.

In the context of dental caries, a dysbiotic shift occurs, characterized by an increased abundance of cariogenic bacteria [141]. *Streptococcus* spp., particularly *Streptococcus mutans*, have been consistently identified as the taxa most strongly associated with caries-active individuals [141,142]. This key etiological agent contributes to the disruption of the balance between demineralization and remineralization through acid production from fermentable carbohydrates, which lowers pH and initiates enamel demineralization [16,142]. Although strongly associated with caries, its relative abundance in the oral microbiome is typically low, indicating that it acts within a broader dysbiotic microbial community rather than dominating it [142]. *Lactobacillus* spp., which are also acidogenic and acid-tolerant, are mainly associated with lesion progression, whereas *S. mutans* are more involved in the early stages of caries development [143,144].

Furthermore, individuals with active caries tend to present greater salivary microbial diversity [145]. Beyond *S. mutans* and *Lactobacillus.*, other microorganisms, including *Streptococcus sobrinus*, *Actinomyces*, *Bifidobacterium* and *Veillonella* have been implicated in the carious process. Emerging evidence also links *Candida albicans* to early childhood caries, reinforcing the contemporary conceptualization of caries as a polymicrobial, dysbiosis-driven disease [146,147].

While the role of dysbiosis in dental caries is well established, whether obesity is associated with a distinct salivary microbial profile remains less clear [63]. Oral bacteria may influence obesity through effects on metabolic efficiency, appetite regulation, and energy metabolism, highlighting a complex interplay that may be circumstantial, opportunistic, or causal [148].

Findings in younger populations are inconsistent. Some studies report no significant differences in *S. mutans*, *Lactobacillus*, overall microbiome richness, or the *Firmicutes*-to-*Bacteroidetes* ratio across different BMI categories [149,150]. In these cases, sex often appears to exert a stronger influence on microbial diversity than BMI [151]. Conversely, other research, including a systematic review [152], suggests that changes in oral microbiota composition and diversity are linked to overweight and obesity in children and adolescents, with *Firmicutes* and *Bacteroidetes* being particularly associated with excess weight [153,154]. Specific bacterial variations have also been observed. *Rothia* spp. may be increased in obese girls and *Neisseria* spp. in obese boys [151], while other studies report a reduced relative abundance of salivary *Veillonella*, *Prevotella*, *Selenomonas*, and *Streptococcus* [155] in obese children.

In adults, more pronounced alterations have been reported, including lower bacterial richness and diversity, alongside shifts in predicted functional pathways [156]. Some researchers have investigated obesity-related differences in salivary composition and observed that obese adults exhibited a higher relative abundance of *Firmicutes* and *Actinobacteria* compared with normal-weight controls [157]. In addition, even though low or undetectable levels of oral *Lactobacillus* have been proposed as a potential marker of susceptibility to future weight gain [158], suggesting a role for specific taxa in body weight regulation, this hypothesis is currently supported by limited evidence and requires independent validation. However, contradictory evidence suggests that in adults with low levels of oral disease, BMI alone may not consistently drive changes in the salivary microbiome when diet and lifestyle are accounted for [159].

Given that both conditions are linked to altered dietary patterns and microbial shifts, it is hypothesized that microbiological changes may underlie the association between dental caries and obesity. Several studies have linked BMI and sugary food consumption to variations in cariogenic bacteria. For instance, obese women consistently show higher salivary counts of *Mutans streptococci* compared with normal-weight peers [61,160]. While habitual sugar intake shows a weak positive relationship with these bacterial counts, the direct association between sweet food consumption and BMI is not always statistically significant, suggesting that lifestyle factors may act as confounders [61,160].

Studies have reported higher levels of *Mutans streptococci* and *Lactobacilli* in obese individuals [153,154] and, accordingly, research in children has shown that BMI is positively associated with both *Mutans streptococci* and *Lactobacilli*, correlating with higher DMFT/dmft scores [119,161].

In overweight and obese adolescents, elevated levels of *S. mutans* and salivary Bifidobacteria have been reported even in caries-free individuals, reinforcing the role of microbial dysbiosis as a precursor to clinical disease [162].

Overall, the available microbiological evidence supports a biological plausibility of a relationship between obesity and dental caries. However, the literature remains insufficiently consistent to support a specific microbial signature shared by both conditions and the available microbiological evidence must be interpreted with caution. The majority of studies are cross-sectional, with varying methodologies and across different age groups, which limits direct comparability. Moreover, few investigations simultaneously control for dietary intake, OH and SES, all of which are strong confounders of both microbial composition and caries risk. As a result, it remains unclear whether the microbial shifts observed in obese individuals represent a cause, a consequence, or merely a correlate of altered oral health status.

Collectively, the evidence synthesized across physicochemical, molecular, and microbial dimensions points toward a coherent, although incompletely characterized, mechanistic framework: obesity-associated metabolic dysregulation appears to produce a salivary environment with reduced ability to neutralize acidic challenges, attenuated immunological defense and increased susceptibility to dysbiotic microbial colonization. A summary of the best available evidence and corresponding strength of evidence, based on consistency of findings and number of available studies, may be found in Table 1. Each of these alterations, even though individually modest in effect, may act synergistically to amplify caries susceptibility in individuals with obesity. Nonetheless, the translational value of current findings remains constrained by considerable methodological heterogeneity and the scarcity of studies simultaneously addressing both conditions and their shared risk factors. This heterogeneity is itself multifactorial, reflecting the lack of standardized obesity definitions and caries indices across studies, differential effects of age and dentition type, geographic and dietary variability and the inconsistent adjustment for socioeconomic confounders. Taken together, it not only limits the synthesis of existing evidence but also underscore the need for methodologically consistent, longitudinal research that simultaneously examines both conditions across diverse populations and life course stages.

## 5. Research Gaps and Future Directions

Despite the growing body of evidence exploring the relationship between obesity and dental caries, significant knowledge gaps remain, particularly regarding the consistency, causality, and clinical applicability of current findings. Addressing these limitations is essential to elucidate the underlying mechanisms of this association and to support the development of robust preventive and diagnostic strategies.

A substantial amount of the available literature consists of observational, predominantly cross-sectional studies, which limits the ability to establish temporal relationships and causal pathways between obesity and dental caries. In addition, interpretation of the available evidence should take age into account, as the biological mechanisms underlying obesity, salivary composition, oral microbial ecology, dietary behaviors, and caries susceptibility may differ substantially between children, adolescents and adults. Future studies should also incorporate sex-stratified analyses, as emerging evidence suggests that biological, microbial, and socioeconomic determinants of the obesity-dental caries relationship may differ between males and females. An additional challenge in this field is the considerable heterogeneity among studies, which may partly explain the conflicting findings regarding the association between BMI and dental caries. Differences in obesity definitions, caries assessment methods and indices, age groups, geographic and cultural contexts, as well as the inconsistent control of socioeconomic and behavioral confounders, limit comparability across studies and highlight the need for standardized research methodologies. Future research should prioritize well-designed longitudinal studies with standardized methodologies and protocols, adequate sample sizes and focusing on the simultaneous evaluation of obesity and dental caries across different age-groups. Furthermore, the current research landscape often prioritizes the discovery of novel biomarkers over the rigorous validation of previously reported ones. Shifting focus toward the replication and cross-validation of existing data is vital for establishing clinical reliability. While salivary biomarkers offer a promising non-invasive window into the biological links between obesity and dental caries, their clinical utility has yet to be fully realized. In the context of obesity, the literature lacks comprehensive validation studies that perform concomitant assessments of salivary biomarkers alongside their corresponding blood concentrations. Furthermore, the integration of high-throughput “omics” technology, such as proteomics and metabolomics, may be required to identify complex salivary signatures that isolated markers fail to capture.

Additionally, with the rapid global adoption of GLP-1 receptor agonists and other weight-loss pharmacotherapies, investigating their specific impact on salivary function and oral health is becoming a clinical priority. Future studies must account for medication-induced changes in the oral environment to avoid bias in obesity-caries assessments.

Finally, establishing a reliable correlation between salivary and systemic measurements is a prerequisite for the integration of saliva-based diagnostics into routine clinical practice. Understanding how weight-related stigma and psychological factors influence healthcare-seeking behaviors will be equally crucial for developing personalized, multidisciplinary intervention programs that address both metabolic and oral health.

## 6. Conclusions

Overall, the relationship between obesity and dental caries is best understood through a multifactorial framework in which shared dietary exposures, OH behaviors and socioeconomic disadvantage constitutes the most consistently supported determinants, while salivary dysfunction and inflammatory shifts represent biologically plausible but less conclusively established contributors. Evidence suggests that this is not a simple direct association, but a complex interplay of shared determinants that unfolds throughout the life course. Current literature does not support a direct, independent causal relationship between obesity and dental caries. Instead, the association is contingent upon age, dentition, and the rigorous control of confounding variables, a finding that strongly reinforces the common risk factor approach. Preventive strategies should therefore shift away from treating obesity and dental caries as isolated conditions and toward integrated interventions targeting their shared determinants.

From a clinical perspective, although prospective evidence evaluating intensified oral health surveillance in obese populations is currently lacking, the available literature suggests that individuals with obesity may represent a group at increased risk of adverse oral health outcomes. Therefore, it is reasonable to consider dietary patterns, oral hygiene practices, and socioeconomic vulnerability as important components of a comprehensive oral health risk assessment, with salivary biomarkers playing a supportive diagnostic role, given the absence of validated panels for routine clinical use. However, even though these biomarkers remain largely investigational, they may emerge as useful non-invasive tools for risk stratification and monitoring in the future. Furthermore, these results highlight the need for multidisciplinary collaboration between dental professionals, nutritionists and primary care physicians to ensure a complete approach to patient health. Finally, further longitudinal research is essential to clarify the temporal sequencing of shared risk exposures, validate salivary biomarker panels against systemic measures and establish whether the microbial and inflammatory shifts observed in obesity precede, accompany or follow the onset of caries activity. Addressing these gaps will provide the scientific foundation necessary for the development of targeted, evidence-based therapeutic approaches, ultimately improving the integrated management of metabolic and oral health.

## Figures and Tables

**Table 1:** Summary of the best available evidence on obesity, dental caries, shared risk factors, and proposed mechanisms linking both conditions.

Topic	Best Available Evidence	Main Findings	Strength of Evidence
Lifestyle and dietary habits	Meta-analysis [57,60,71] Systematic review [42,43,47,58,70,72]	↑ Free sugars and SSB = ↑ BMI and ↑ caries scores; Frequency and timing of intake = critical modifiers; Family, educational and environmental factors strongly influence dietary behaviors; Tooth loss and impaired masticatory function: may contribute to the obesity-caries relationship.	Strong
Oral hygiene habits	Meta-analysis [73,80] Systematic review [74] Systematic Analysis [75]	Brushing frequency and caries = inverse association; Poor OH may be associated with ↑ BMI OH practices may partially mediate the saliva-caries association in obesity; however evidence is scarce.	Moderate
Socioeconomic status (income, education, occupation)	Meta-analysis [26,90,94,95,96] Systematic review [91,97,98]	Low SES: ↑ risk of both conditions; SES influences dietary behaviors, access to care and health literacy; Findings differ across income-level settings.	Strong
Physicochemical properties (pH, flow rate, buffering capacity)	Meta-analysis [18,60,106,110] Systematic review [101]	Obesity: ↓ flow rate, ↓ pH, ↓ buffering capacity; Physicochemical changes = ↑ caries susceptibility; Physicochemical changes may partially contribute to the obesity-caries association but do not fully explain it.	Moderate
Molecular composition (TNF-α, IL-6, IL-8, IgA, α-amylase, TAC)	Meta-analysis [18,100,105,107,123,129] Systematic review [102,115,116,118]	TNF-α and IL-6: show the most consistent associations with both conditions individually; IgA and α-amylase: dual and context-dependent; Simultaneous evaluation of both conditions is scarce; Insufficient evidence to support clinical use as diagnostic/prognostic tools in obesity-caries relationship.	Limited to Moderate
Microbial profile	Meta-analysis [142,147] Systematic review [152]	Dental caries: associated with oral microbial dysbiosis, particularly ↑ *S. mutans* and ↑ *Lactobacillus* spp.; Obesity: may be associated with altered salivary microbiome; findings remain inconsistent; Microbial dysbiosis may contribute to the obesity-caries association; current evidence remains inconclusive.	Limited

Note: Strength of evidence was assigned narratively according to the consistency of findings, the availability of systematic reviews and meta-analyses, and the overall quality and quantity of the available evidence. Strong: consistent findings supported by multiple high-quality studies; Moderate: generally consistent findings with some limitations or heterogeneity; Limited: scarce and inconsistent evidence.

## Data Availability

No new data were created or analyzed in this study.

## Abbreviations

The following abbreviations are used in this manuscript:

WHO	World Health Organization
BMI	Body Mass Index
SES	Socioeconomic Status
OH	Oral Hygiene
DMFT	Decayed, Missing and Filled Teeth (permanent teeth)
dmft	decayed, missing and filled teeth (deciduous teeth)
SSB	Sugar-sweetened Beverages
GLP-1	Glucagon-like Peptide-1
TNF-α	Tumor Necrosis Factor-alpha
α-amylase	Alpha-amylase
TAC	Total Antioxidant Capacity
IL	Interleukin
sAA	Salivary Alpha-amylase
IgA	Immunoglobulin A
